# Trajectories of detailed general movements and their association with one-year developmental outcomes in very preterm, moderate-to-late preterm, and term infants

**DOI:** 10.1007/s00431-026-06971-x

**Published:** 2026-05-01

**Authors:** Doğan Porsnok, Bilge Nur Yardımcı-Lokmanoğlu, Akmer Mutlu

**Affiliations:** 1 Faculty of Physical Therapy and Rehabilitation, Developmental and Early Physiotherapy Unit, Hacettepe University, Ankara, Turkey; 2 Faculty of Physical Therapy and Rehabilitation, Bingöl University, Bingöl, Turkey

**Keywords:** Early spontaneous movements, Developmental outcome, General movements, Optimality score, Preterm infants

## Abstract

Trajectories of early spontaneous movements provide valuable insights into developmental outcomes in preterm infants. We aimed to compare the longitudinal trajectory of early spontaneous movements in very preterm (VPT), moderate-to-late preterm (MLP) and term infants, and determine the relationship between early spontaneous movements and one-year developmental outcomes in preterm infants. This prospective longitudinal study included 133 infants, comprising 91 preterm and 42 term infants. Early spontaneous movements were assessed longitudinally according to the infants’ ages, using the revised General Movements Assessment (GMA) score sheet, the General Movements Optimality Score-Revised (GMOS-R), and the Motor Optimality Score for 3-to 5-Month-Old Infants-Revised (MOS-R). Developmental outcomes were assessed using the Bayley Scales of Infant and Toddler Development-Third Edition (Bayley-III). Two hundred and twenty-four video recordings for GMOS-R and 133 video recordings for MOS-R were evaluated. There was no difference in GMOS-R between VPT and MLP infants in the preterm or term periods (*p* = 0.343). However, VPT infants exhibited lower GMOS-R (*p* = 0.001) in the postterm period and lower MOS-R (*p* = 0.018) in the fidgety period compared to term infants. VPT infants had lower Bayley-III outcomes than MLP infants in the cognitive (p = 0.006), language (p = 0.003), and motor domains (p = 0.004), and lower motor outcome than term infants (p < 0.001). GMOS-R in the preterm or term periods was correlated only with motor outcomes (*p* = 0.002; *r* = 0.329), while in the postterm period GMOS-R was correlated with cognitive (*p* = 0.039; *r* = 0.222), language (*p* = 0.045; *r* = 0.215), and motor (*p* = 0.001; *r* = 0.340) outcomes in all preterm infants. MOS-R was also related with cognitive (*p* < 0.001; *r* = 0.396), language (*p* = 0.005; *r* = 0.301), and motor (*p* < 0.001; *r* = 0.457) outcomes.

*Conclusion*: This study supports the concept that VPT infants are at high-risk of developmental problems. Additionally, while the relationship of GMOS-R to developmental outcomes is highlighted, MOS-R is more strongly associated with later developmental outcomes.

**Table:** 

**What is Known:** • *Time-based General Movements Assessment through trajectories provide important information to identify infants at high neurodevelopmental risk.* **What is New:** • *The General Movements Optimality Score-Revised (GMOS-R) may support early risk stratification in the earliest months of life and is potentially informative for early intervention referrals. However, MOS-R demonstrates stronger overall associations, highlighting its important role in early developmental assessments.*

**Supplementary information:**

The online version contains supplementary material available at 10.1007/s00431-026-06971-x.

## Introduction

Preterm infants are defined as infants born < 37 weeks of gestation, with more than 1 in 10 live births worldwide [1, 2]. Preterm birth causes high mortality and morbidity rates, and many of the preterm infants had lower motor, cognitive, and academic performance compared with term children [1, 3–5]. Early identification of risk for disabilities, including cerebral palsy (CP) and other long-term adverse effects, might enable early intervention and achieve better development outcomes in children—as well as enhance parent and caregiver well-being [6, 7]. Early intervention may allow the introduction of high-potential neuroplasticity in the earliest period [6]. It has been reported that early intervention is possible with early identification by using assessment tools that have high predictive validity before 5 months of age [7]. The General Movement Assessment (GMA) is one of the most effective predictive assessments for the development of CP, along with magnetic resonance imaging (MRI) and the Hammersmith Infant Neurological Examination (HINE) [7].

General Movements (GMs) are spontaneous movements that emerge in the whole body from the postmenstrual 9 weeks to the postterm 20 weeks and provide insights on the integrity of the developing nervous system [8, 9]. GMs are generated by central pattern generators (CPGs) located in the brainstem which generate rhythmic motor patterns. They are called preterm GMs before the term period, and referred to as writhing movements from the term period up to the first 8 postterm weeks of life [8, 10]. GMs change appearance due to the ongoing neural transition—these are called fidgety movements from 9 weeks up to 20 weeks postterm age [8, 11, 12]. The complexity and variability of GMs decline when impairment in the nervous system is present [8, 9, 13]. Abnormal GMs in the preterm and term periods are classified as poor repertoire (PR) GMs, cramped synchronized (CS) GMs, chaotic (Ch) GMs, and classified as absent fidgety movements or abnormal fidgety movements between 9 and 20 weeks postterm age [8].

In addition to global assessment, a detailed GMA has been developed: General Movements Optimality Score (GMOS) [8, 14] or the recently published revised version, the General Movements Optimality Score-Revised (GMOS-R) (15), for the preterm and term period until 8 weeks, and Motor Optimality Score (MOS) [8] or Motor Optimality Score-Revised (MOS-R) [16] for the fidgety period between 9 and 20 weeks postterm age. The GMOS provides semi-quantification of GMs quality by scoring parameters of movement, such as amplitude, speed, range, onset, and offset [14]. Einspieler et al. [14] found that the GMOS results for normal and PR GMs at postterm age (≥ 42 to 45 weeks) were slightly lower than at preterm (< 37 weeks) and term age (≥ 37 to 42 weeks). Barbosa et al. [17] also reported that GMOS results separated typical development from atypical development successfully and CP from other neurodevelopmental disorders, based on specific clinical features, including age, type of brain injury and clinical outcomes at 2 years of age. A pilot study by Yin et al. [18] showed that the mean GMOS at the ipsilesional limb was higher than the contralesional limb, indicating that GMOS could successfully detect and separate asymmetric movements in infants with neonatal arterial ischemic stroke who are at high-risk of developing unilateral CP. Preterm infants had low MOS compared to term-born peers [19, 20]; low MOS is associated with poor gross and fine motor performance at 12-months [21], low performance for general cognition, attention, working memory, executive function, motor function at 8 years [22], minor neurologic dysfunction at 7–11 [23] and poor cognitive outcomes in young adulthood [24].

Time-based GMA through trajectories provides important information for identifying infants at high neurodevelopmental risk [25–27]. Although there are studies which include trajectories in infants born preterm, [14, 27–31] the majority have generally focused on the global GMA results [27, 29–31]. There is a need to expand studies on the trajectories of preterm infants using detailed GMA that includes both GMOS-R and MOS-R. Moreover, most of these studies have been conducted on high-risk groups, such as those with a gestational age of less than 32 weeks or with a very low birth weight [27, 30, 31]. It is well known that the risk of adverse developmental outcomes increases with decreasing gestational age [32, 33], but moderate-late preterm infants are considered to be at risk for developmental problems [34, 35]. Therefore, it is clinically important to provide all outcomes for all premature infants from an early stage, without overlooking any of them.

This study aimed (i) to perform a longitudinal examination of the changes in early spontaneous movements, (ii) compare the longitudinal trajectory of early spontaneous movements between different gestational age groups, such as very preterm (VPT), moderate to late preterm (MLP), and term infants, and (iii) investigate cognitive, language and motor development in these infants at one year of age and the relationship between early spontaneous movements and developmental outcomes in preterm infants.

## Methods

### Participants and procedure

**Fig. 1:**
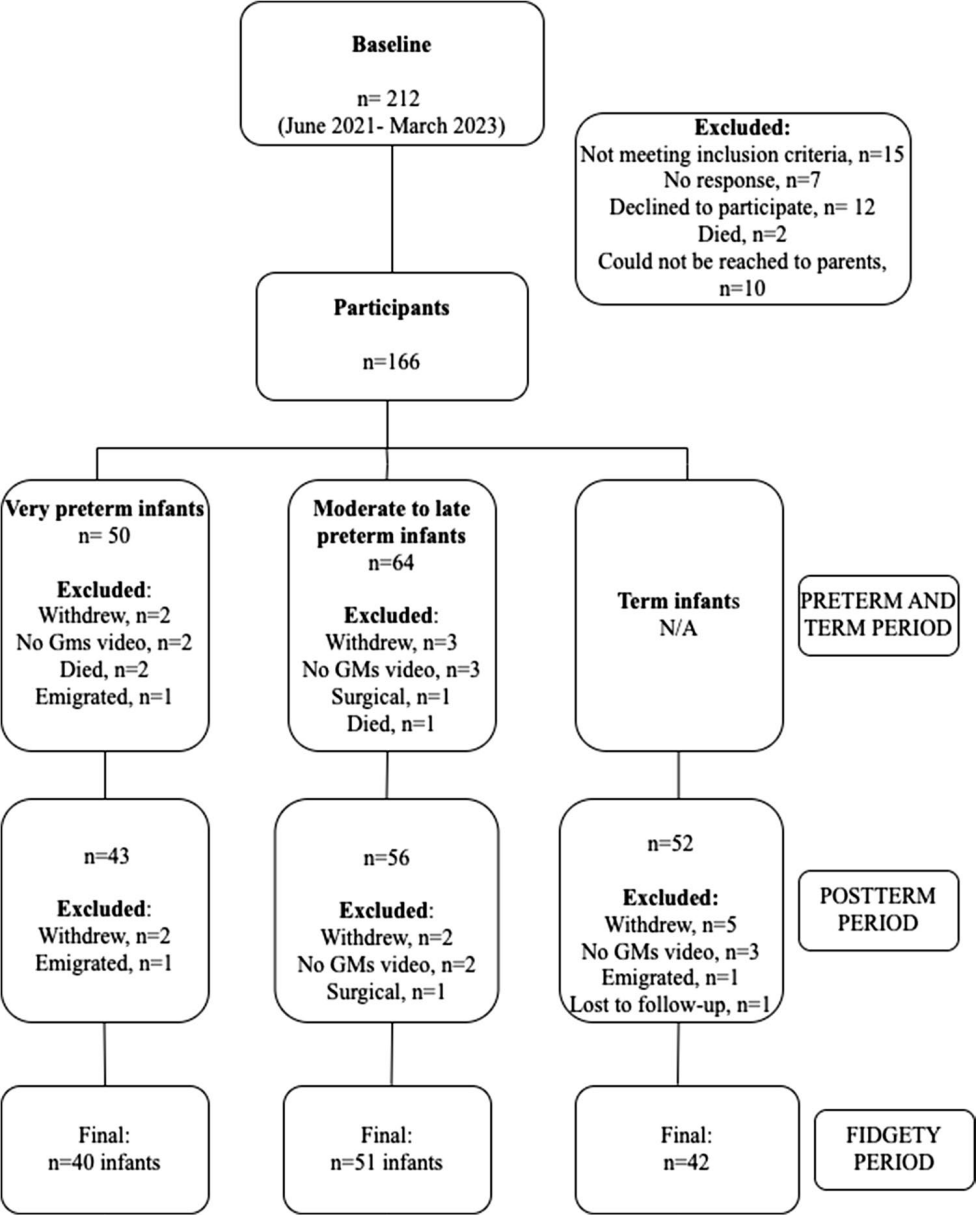
Flowchart of this study

This prospective study included preterm and term infants who applied to the Developmental and Early Physiotherapy Unit at Hacettepe University from June 1, 2021 to March 1, 2023. Preterm infants were included in the study if they were referred to our clinic before 37 weeks of gestation. Exclusion criteria were: (i) having any major congenital or chromosomal anomaly, (ii) having undergone a surgical operation such as for congenital heart defects, (iii) not having participated in any of the evaluation periods of GMA, and (iv) family’s refusal to participate in the study (Fig. 1). All preterm infants were consecutively included in the study, except for those who met the predefined exclusion criteria. Term infants, as a control group, consisted of those referred to our clinic for developmental follow-up due to transient and low-risk neonatal conditions, such as hyperbilirubinemia, hypoglycemia, and irregular breathing patterns. Term infants with any neurological condition were excluded from the study. GMA was performed on preterm infants at three different time points based on postmenstrual age: (1) preterm period (34 to 36 weeks) or term period (37 to 41 weeks), (2) postterm period (42 to 45 weeks), and (3) fidgety period (49 to 60 weeks) after they were referred to our clinic. Term infants were assessed twice—postterm period (42 to 45 weeks) and fidgety period (49 to 60 weeks). A total of one hundred and thirty-three infants included 40 very preterm infants (VPT infants, < 32 weeks of gestation), 51 moderate to late preterm infants (MLP infants, ≥ 32 to < 37 weeks of gestation), and 42 term infants (≥ 37 to < 42 weeks of gestation). The clinical characteristics of all infants are presented in Table 1. The study was approved by the Non-interventional Ethical Review Board of Hacettepe University (decision number GO 21/448, dated 06.04.2021), and all parents provided written informed consent.

**Table 1:** Infant characteristics

	VPT infants, *n* = 40	MLP infants, *n* = 51	Term infants, *n* = 42	*p*	Post-hoc p
Male, *n* (%)	18 (45)	22 (43.1)	21 (50)	0.797^a^	—
*Gestational age (weeks)*					
Median	30	35	38	** < 0.001**^**c**^	**VPT vs MLP: < 0.001**^**b**^ **VPT vs term: < 0.001**^**b**^ **MLP vs term: < 0.001**^**b**^
Range	26–32	32–36	37–40		
*Birth weight (g)*					
Median	1145 g	2520	2995	** < 0.001**^**c**^	**VPT vs MLP: < 0.001**^**b**^ **VPT vs term: < 0.001**^**b**^ **MLP vs term: < 0.001**^**b**^
Range	750–2100	1470–3200	2490–4040		
< 10th percentile, *n* (%)	3 (7.5)	2 (3.9)	0 (0)	0.079^a^	—
10th-90th percentile, *n* (%)	33 (82.5)	47 (92.2)	42 (100)		
> 90th percentile, *n* (%)	4 (10)	2 (3.9)	0 (0)		
Intrauterine growth restriction *n* (%)	5 (12.5)	3 (5.8)	0 (0)	0.059^a^	—
Intraventriculer hemorrhage, *n* (%)	10 (25)	0 (0)	0 (0)	** < 0.001**^**a**^	**VPT vs MLP: 0.002**^**a**^ **VPT vs term: 0.008**^**a**^
Grade 1–2, *n* (%)	8 (20)	0 (0)	0 (0)		
Grade 3–4, *n* (%)	2 (5)	0 (0)	0 (0)		
Periventricular leukomalacia, *n* (%)	1 (2.5)	0 (0)	0 (0)	0.310^a^	—
Bronchopulmonary dysplasia, *n* (%)	17 (42.5)	0 (0)	0 (0)	** < 0.001**^**a**^	**VPT vs MLP: < 0.001**^**a**^ **VPT vs term: < 0.001**^**a**^
Patent ductus arteriosus, *n* (%)	23 (57.5)	4 (7.8)	1 (2.4)	** < 0.001**^**a**^	**VPT vs MLP: < 0.001**^**a**^ **VPT vs term: < 0.001**^**a**^
Respiratory distress syndrome, *n* (%)	23 (57.5)	1 (2)	0 (0)	** < 0.001**^**a**^	**VPT vs MLP: < 0.001**^**a**^ **VPT vs term: < 0.001**^**a**^
Retinopathy of prematurity, *n* (%)	31 (77.5)	0 (0)	0 (0)	** < 0.001**^**a**^	**VPT vs MLP: < 0.001**^**a**^ **VPT vs term: < 0.001**^**a**^
Hyperbilirubinemia, *n* (%)	1 (2.5)	12 (23.5)	2 (4.8)	**0.001**^**a**^	VPT vs MLP: 0.017^a^ **MLP vs term: 0.006**^**a**^
Necrotizing enterocolitis, *n* (%)	1 (2.5)	0 (0)	0 (0)	0.310^a^	—
Prenatal maternal health problems (%)					
Preeclampsia, *n* (%)	9 (22.5)	7 (13.7)	3 (7.1)	0.138^a^	—
Gestational diabetes, *n* (%)	6 (15)	9 (17.6)	11 (26.2)	0.402^a^	—

AGA = appropriate for gestational age, MLP = moderate-to-late preterm, VPT = very preterm

Bold values indicate statistically significant at the *p* < 0.05 level. Post-hoc pairwise comparisons were adjusted using Bonferroni correction (*p* < 0.017). Bold values indicate statistically significant results after correction

^a^Pearson chi-square test

^b^Mann-Whitney *U* test

^c^Kruskal-Wallis test

### Evaluation of the prechtl general movements assessment (GMA)

All infants were videotaped for between 3 and 5 min as a part of GMA. In total, the preterm infants (VPT/MLP infants) were videotaped three times, and the term infants twice—as mentioned above. All video recordings were performed according to Prechtl GMA standards [8]. Two scorers (A.M and B.N.Y.L) who are certified with basic and advanced GMA level, and were unaware of the infants’ medical history and clinical findings, analyzed the videos. In case of disagreement in the scoring of global and detailed GMA (6 recordings at the preterm or term periods, 6.5%; 8 recordings at the postterm period, 6%; and 4 recordings at the fidgety period, 3%), the scorers discussed the recording until consensus was achieved. The intraclass correlation coefficient between the observers for GMOS-R and MOS-R in our study was 0.93 (95% confidence interval = 0.87 to 0.96).

### General movement optimality score-revised (GMOS-R)

GMOS-R evaluates the movement quality of the neck, trunk, lower and upper extremities separately and provides an optimality score as well as the global GMA including normal GMs, PR GMs, CS GMs, and Ch GMs [15]. The GMOS-R score sheet comprised the following subcategories: (i) the sequence of movement (maximum 2 points), (ii) neck and trunk (maximum 4 points), (iii) upper extremities (maximum 16 points), and (iv) lower extremities (maximum 16 points) [15]. The score ranges from 0 to 38; while 38 indicates optimal motor performance, 0 indicates the worst motor performance [15].

### Motor optimality score-revised (MOS-R)

The detailed GMA assessed not only fidgety movements but also other movement and postural patterns using the revised score sheet of the Motor Optimality Score for 3- to 5-Month-Old-Infants–Revised [16], from which the MOS-R is determined. The MOS-R score sheet consists of 5 subcategories: (i) temporal organization and quality of fidgety movements (maximum 12 points), (ii) observed movement patterns (maximum 4 points), (iii) age-adequate movement repertoire other than fidgety movements (maximum 4 points), (iv) observed postural patterns and (v) movement character (maximum 4 points). The MOS-R ranges from 5 (low optimality) to 28 (high optimality) [8, 16]. MOS-R of 5–8 was classified as severely reduced, 9–19 as moderately reduced, 20–24 as mildly reduced and 25–28 as optimal [16].

#### Evaluation of developmental outcomes at 1 year of age

The developmental outcomes of infants, including cognitive, language, and motor outcomes, were assessed using the Bayley Scales of Infant and Toddler Development, 3rd edition (Bayley-III) at 1 year of age. Bayley-III was administered by certified evaluators blinded to GMA results. In the Bayley-III, corrected age was used for preterm infants—calculated by subtracting weeks of prematurity from chronological age—while chronological age was applied for term infants. A composite score was calculated, which was used to categorize subjects as having average, mild, or moderate to severe impairment. A composite score of 100, with a standard deviation (SD) of 15, is indicative of average functioning. In the Bayley-III domains, a composite score below 85 was indicated as developmental delay. While a composite score of 70 to 85 (1 SD below the mean) was considered indicative of “mild impairment,” a score of < 70 (> 2 SD below the mean) was classified as “moderate to severe impairment” [36].

#### Statistical analysis

The SPSS package for Macintosh, version 25.0 (SPSS Inc, Chicago, IL, USA), was used for statistical analysis. *P*-values of less than 0.05 were considered significant for all tests employed. Nonparametric tests were used when the variables were not normally distributed. The Pearson chi-square test or Fisher’s exact test were used to compare categorical variables, while the Mann–Whitney *U* and Kruskal–Wallis tests were used to compare GMOS-R, MOS-R, gestational age, and birth weight as well as other ordinal variables. The Mann–Whitney *U* test and chi-square test were conducted to evaluate the significance of pairwise differences—using the Bonferroni correction to adjust for multiple comparisons. The Bonferroni correction used for post-hoc pairwise comparisons had a statistical significance level of *p*** < **0.017. The effect size was assessed using the epsilon-squared statistic (ε^2^) (≥ 0.01 = small effect; ≥ 0.06 = moderate effect; ≥ 0.14 = large effect) for the Kruskal–Wallis analyses, while the formula *r* = Z/√N (0.10 ≤ *r* < 0.30 = small effect; 0.30 ≤ *r* < 0.50 = moderate effect; *r* ≥ 0.5 = large effect) was used for the effect size in the Mann–Whitney *U* analyses [37]. We analyzed the relationships between early spontaneous movements and Bayley-III using the Spearman correlation coefficient; ranging from 0.90 to 1.00 indicating very high correlation; 0.70 to 0.89 indicating strong correlation; 0.50 to 0.69 indicating moderate correlation; 0.30 to 0.49 indicating low correlation; and 0.00 to 0.29 indicating little to no correlation [38]. Correlation analyses were performed as exploratory analyses to assess potential correlations among variables. Furthermore, an attrition analysis was conducted on birth weight, gestational age, GMOS-R and MOS-R to explore the possible effects of missing data among infants who either completed or did not complete the Bayley-III. The missing Bayley-III data were deemed to be missing at random (MAR) based on attrition analyses, so available case analysis was used. A multivariable linear regression analysis was performed to evaluate the relationship between the potential predictors and the Bayley-III motor composite score. Due to their clinical relevance associated with motor outcomes, the MOS-R, gestational age, intraventricular hemorrhage (IVH) grade III–IV (yes/no), and bronchopulmonary dysplasia (BPD, yes/no) were included into the model.

## Results

All clinical characteristics and demographic information of all infants are presented in Table 1. We examined 357 video recordings of 133 infants, 91 in the preterm or term periods, 133 in the postterm period, and 133 in the fidgety period.

### Longitudinally early spontaneous movements

A total of 91 video recordings were assessed at the preterm or term periods. The majority of global GMs recordings were scored as PR (*n* = 61, 67%), 21 as normal GMs (23%), and 9 CS GMs (10%). The median GMOS-R was 31 (ranging from 28 to 35) for normal GMs, 20 for PR GMs (ranging from 9 to 32), and 12 for CS GMs (ranging from 7 to 17). In total, 133 video recordings were also assessed at the postterm period. The majority of global GMs recordings were scored as PR GMs (*n* = 90, 67%). There were 37 normal GMs (28%), and 6 CS GMs (5%). The median GMOS-R was 32 (ranging from 28 to 38) for normal GMs, 26 for PR GMs (ranging from 11 to 31), and 13.5 for CS GMs (ranging from 8 to 17).

**Fig. 2:**
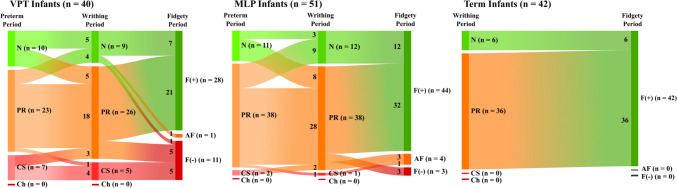
Longitudinal general movements trajectory of VPT, MLP and term infants. MLP, moderate-to-late preterm; VPT, very preterm, N, normal; PR, poor repertoire; CS, cramped synchronized; Ch, chaotic

A total of 133 video recordings were assessed during the fidgety period. One hundred and fourteen infants (85.7%) had normal fidgety movements, 14 infants (10.5%) displayed absent fidgety movements, and five infants (3.8%) showed abnormal or exaggerated fidgety movements (Fig. 2). The MOS-R ranged from 6 to 28, with a median = 24. MOS-R was optimal in 60 infants (45.1%), mildly reduced in 52 infants (39.1%), moderately reduced in 19 infants (14.3%), and severely reduced in 2 infants (1.5%).

### Early spontaneous movements at the preterm or term periods (VPT/MLP infants)

There was no significant difference in GMOS-R (*p* = 0.343) and global assessment (*p* = 0.072) between VPT and MLP infants. The median GMOS-R was 21 in VPT infants and 23 in MLP infants, and the most common GMs pattern was found to be PR GMs in both VPT and MLP infants. The median of all other subcategories of GMOS-R was also similar between groups; sequence (*p* = 0.566), neck and trunk (*p* = 0.639), upper extremities (*p* = 0.585), and lower extremities (*p* = 0.140) (Table 2, Fig. 2).

**Table 2:** General Movements Optimality Score-Revised (GMOS-R) at preterm or term and postterm periods

	Preterm or term period			Postterm period				
	VPT infants, *n* = 40	MLP infants, *n* = 51	*p*	VPT infants, *n* = 40	MLP infants, *n* = 51	Term infants, *n* = 42	*p*	Post-hoc p
Recording age, wks, median (IQR)	38 (36.2–39)	38 (37–38)	0.664^b^	44 (43–44.75)	43 (43–44)	44 (43–44)	0.466^c^	—
Global assessment								
N (%)	10 (25)	11 (21.6)	0.072^a^	9 (22.5)	16 (31.4)	12 (28.5)	0.059^a^	—
PR (%)	23 (57.5)	38 (74.5)		26 (65)	34 (66.7)	30 (71.4)		
CS (%)	7 (17.5)	2 (3.9)		5 (12.5)	1 (2)	0 (0)		
Ch (%)	0 (0)	0 (0)		0 (0)	0 (0)	0 (0)		
Sequence, median (IQR) mean (SD)	1 (1–1.75) 1.075 (0.65)	1 (1–1) 1.15 (0.50)	0.566^b^	1 (1–1) 1.10 (0.59)	1 (1–2) 1.27 (0.53)	1 (1–2) 1.28 (0.45)	0.267^c^	—
Neck and trunk OS, median (IQR) mean (SD)	1 (0.25–2) 1.40 (1.1)	1 (1–2) 1.50 (1.06)	0.639^b^	2 (1–2) 1.47 (1.03)	2 (1–2) 1.76 (0.92)	2 (1–2) 1.85 (0.81)	0.197^c^	—
Upper extremities OS, median (IQR) mean (SD)	10 (7–13) 9.97 (3.26)	10 (8–13) 10.35 (3.16)	0.585^b^	11 (8–13) 10.70 (3.21)	13 (10 −14) 11.94 (2.47)	13.5 (12–14) 12.97 (2.15)	**0.002**^**c**^** (ε**^**2**^** = 0.097)**	**VPT vs term: 0.001**^**b**^ MLP vs term: 0.036^b^ VPT vs MLP: 0.057^b^
Lower extremities OS, median (IQR) mean (SD)	8 (6–12) 8.45 (3.78)	10 (7–12) 9.60 (3.25)	0.140^b^	11 (7–13) 10.15 (3.64)	12 (10–14) 11.86 (2.48)	13 (11–14) 12.42 (2.08)	**0.009**^**c**^** (ε**^**2**^** = 0.071)**	**VPT vs term: 0.004**^**b**^ MLP vs term: 0.369^b^ VPT vs MLP: 0.024^b^
GMOS-R, median (IQR) mean (SD)	21 (13.25–29) 20.92 (8.07)	23 (16–28) 22.62 (6.94)	0.343^b^	25 (17.25–29) 23.42 (7.47)	28 (23–31) 26.76 (5.33)	28.5 (25–31) 28.42 (4.04)	**0.004**^**c**^** (ε**^**2**^** = 0.082)**	**VPT vs term: 0.001**^**b**^ MLP vs term: 0.230^b^ VPT vs MLP: 0.024^b^
Age-Specific GMOS-R Centile Rank, median (IQR) mean (SD)	55.2 (34.8–76.3 55.0 (21.3)	60.5 (42.1–73.6) 59.5 (18.3)	0.343^b^	65.7 (45.3–76.3) 61.6 (19.7)	73.6 (60.5–81.5) 70.4 (14.1)	74.9 (67.7–81.5) 74.9 (10.5)	**0.004**^**c**^** (ε**^**2**^** = 0.082)**	**VPT vs term: 0.001**^**b**^ MLP vs term: 0.230^b^ VPT vs MLP: 0.024^b^

GMOS-R = General Movement Optimality Score–Revised, MLP = moderate-to-late preterm, VPT = very preterm, IQR = interquartile range, NA = not applicable, OS = Optimality Score, SD = standard deviation, N = normal, PR = poor repertoire, CS = cramped synchronized, Ch = chaotic

ε^2^ = epsilon-squared effect size (≥ 0.01 = small effect; ≥ 0.06 = moderate effect; ≥ 0.14 = large effect)

Bold values indicates statistically significant at the *p* < 0.05 level. Post-hoc pairwise comparisons were adjusted using Bonferroni correction (*p* < 0.017). Bold values indicate statistically significant results after correction

^a^Pearson chi-square test

^b^Mann-Whitney *U* test

^c^Kruskal-Wallis Test

#### Early spontaneous movements at postterm period (VPT/MLP/term infants)

There were significant differences in GMOS-R between three groups (*p* = 0.004; *ε*^2^ = 0.082). After the Bonferroni correction, it was determined that VPT infants had lower GMOS-R than term infants (*p* = 0.001; *r* = 0.36). In pairwise comparisons, a difference between VPT and MLP infants was observed at the unadjusted level (*p*** = **0.024). However, this did not remain statistically significant after Bonferroni correction. The median GMOS-R for VPT infants was 25, for MLP infants 28, and for term infants 28.5. The global assessment results between groups did not differ (*p* = 0.059). The most common GMs pattern was also found to be PR GMs in all groups. There was a significant difference in optimality scores of upper extremities (*p* = 0.002; *ε*^2^ = 0.097), and lower extremities (*p* = 0.009; *ε*^2^ = 0.071) between VPT, MLP, and term infants. There were no significant differences between optimality scores of the other GMOS-R subcategories; sequence (*p* = 0.267), neck and trunk (*p* = 0.197) (Table 2, Fig. 2).

### Early spontaneous movements at the fidgety period (VPT/MLP/term infants)

There were significant differences in MOS-R results (*p* = 0.047; *ε*^2^ = 0.046) and fidgety movements (*p* = 0.001) between all groups. The median MOS-R was 24 for VPT infants, 24 for MLP infants, and 25 for term infants. When the Bonferroni correction was performed, it was found that VPT infants had lower MOS-R than term infants (*p* = 0.018; *r* = 0.26). All term infants had normal fidgety movements, compared to 86.3% in the MLP infants, and 70% in the VPT infants. The normal fidgety movements rate was found to be significantly higher in both the term (*p* = 0.001) and MLP (*p* = 0.013) infants compared to the VPT infants. Differences are also observed in movement patterns (*p* = 0.048) (Table 3, Fig. 2).

**Table 3:** Motor Optimality Score of VPT, MLP and term infants

	VPT infants, *n* = 40	MLP infants, *n* = 51	Term infants, *n* = 42	Comparison between groups	Post-hoc p
Recording age (weeks), median (IQR)	53 (52–54)	53 (52–53)	52 (52–53)	0.052	—
MOS-R score, median (IQR) Mean (SD)	24 (13.25–26) 20.4 (7.2)	24 (22–28) 23.6 (4.7)	25 (24–26) 25.07 (1.8)	**0.047**^**c**^ **(ε**^**2**^** = 0.046)**	VPT vs MLP: 0.066^b^ **VPT vs term: 0.018**^**b**^ MLP vs term: 0.496^b^
Range	6–28	9–28	21–28	-	
Severely reduced (5–8) (%)	2 (5)	0 (0)	0 (0)	**0.002**^**a**^	VPT vs MLP: 0.075^a^ **VPT vs term: < 0.001**^**a**^ MLP vs term: 0.044^a^
Moderately reduced (9–19) (%)	12 (30)	7 (13.7)	0 (0)		
Mildly reduced (20–24) (%	11 (27.5)	21 (41.2)	20 (47.6)		
Optimal (25–28) (%)	15 (37.5)	23 (45.1)	22 (52.4)		
Fidgety movements					
Normal, *n* (%)	28 (70)	44 (86.3)	42 (100)	** < 0.001**^**a**^	**VPT vs MLP: 0.013**^**a**^ **VPT vs term: 0.001**^**a**^ MLP vs term: 0.044^a^
Abnormal, *n* (%)	1 (2.5)	4 (7.8)	0 (0)		
Absent/sporadic, *n* (%)	11 (27.5)	3 (5.9)	0(0)		
Observed movement patterns					
N > A, *n* (%)	36 (90)	51 (100)	42 (100)	**0.048**^**a**^	VPT vs MLP: 0.069^a^ VPT vs term: 0.110^a^ MLP vs term: 1.000^a^
N = A, *n* (%)	2 (5)	0 (0)	0 (0)		
N < A, *n* (%)	2 (5)	0 (0)	0 (0)		
Age-adequate movement repertoire					
Present, *n* (%)	14 (35)	27 (51.9)	21 (50)	0.396^a^	—
Reduced, *n* (%)	14 (35)	16 (31.4)	12 (28.6)		
Absent, *n* (%)	12 (30)	8 (15.7)	9 (21.4)		
Postural patterns					
N > A, *n* (%)	23 (57.5)	35 (68.6)	36 (85.7)	0.073^a^	—
N = A, *n* (%)	9 (22.5)	10 (19.6)	4 (9.5)		
N < A, *n* (%)	8 (20)	6 (11.8)	2 (4.8)		
Movement character					
Smooth and fluent, *n* (%)	12 (30)	19 (37.3)	12 (28.6)	0.522^a^	—
Abnormal, not CS, *n* (%)	27 (67.5)	32 (62.7)	30 (71.4)		
CS, *n* (%)	1 (2.5)	0 (0)	0 (0)		

*MLP* moderate-to-late preterm, *VPT* very preterm, *CS* cramped-synchronized, *IQR* interquartile range, *MOS-R* Motor Optimality Score-Revised, *N > A* more normal than abnormal patterns, *N = A* an equal number of normal and abnormal patterns, *N < A* fewer normal than abnormal patterns, *SD* standard deviation

ε^2^ = epsilon-squared effect size (≥ 0.01 = small; ≥ 0.06 = moderate; ≥ 0.14 = large)

Bold values indicates statistically significant at the *p* < 0.05 level. Post-hoc pairwise comparisons were adjusted using Bonferroni correction (*p* < 0.017). Bold values indicate statistically significant results after correction

^a^Pearson chi-square test

^b^Mann-Whitney *U* test

^c^Kruskal-Wallis test

## Bayley-III at one year of age

There were differences between the cognitive (*p* = 0.014; *ε*^2^ = 0.071), language (*p* = 0.008; *ε*^2^ = 0.081) and motor (*p* = 0.001; *ε*^2^ = 0.121) domains of VPT, MLP and term infants at 1 year of age. MLP infants had higher results in cognitive, language and motor domains than VPT infants, and term infants had higher motor domain results than VPT infants, with the Bonferroni correction. Table 4 shows the rates of motor, cognitive and language impairment, and the median Bayley-III outcomes. In preterm infants, the GMOS-R at the preterm or term periods showed a positive correlation with the Bayley-III motor domain (*p* = 0.002, *r* = 0.329), while the postterm GMOS-R also showed a correlation with the cognitive (*p* = 0.039, *r* = 0.222), language (*p* = 0.045, *r* = 0.215), and motor (*p* = 0.001, *r* = 0.340) domains. In addition, the global GMA results showed a weak correlation with the motor domain (*p* = 0.017, *r* = 0.255) in the preterm or term periods and a moderate association with the cognitive (*p* = 0.001, *r* = 0.355) and motor (*p* = 0.001, *r* = 0.363) domains in the postterm period. Furthermore, the MOS-R showed a positive correlation with the cognitive (*p* < 0.001, *r* = 0.396), language (*p* = 0.005, *r* = 0.301), and motor domains (*p* < 0.001, *r* = 0.457). The fidgety movements subcategory also exhibited moderate correlation with the motor domain (*p* = 0.016, *r* = 0.451). For MOS-R, analyses subtracting the fidgety movements subcategory showed moderate correlation with the cognitive (*p* < 0.001, *r* = 0.393) and motor domains (*p* < 0.001, *r* = 0.394) (Supplementary Table 1). The missingness rates of the Bayley-III assessment differed between the VPT, MLP, and term infants (*p* = 0.045). However, when each gestational group was evaluated separately, comparing infants who completed the Bayley-III with those who did not complete it revealed no significant differences in basic demographic characteristics, such as gestational age (*p* = 0.051–0.937), birth weight (*p* = 0.226–0.937) and sex (*p* = 0.181–0.697), or in early spontaneous movements GMOS-R (*p* = 0.127–0.831) and MOS-R (*p* = 0.177–0.615).

**Table 4:** Bayley-III results at 1 year of age

Bayley-III Scores	VPT infants, *n* = 38	MLP infants, *n* = 49	Term infants, *n* = 34	Comparison between groups	Post-hoc p
Assessment age (months), median (IQR)	12 (12–13)	12.5 (12–12.5)	12 (12–12.5)	0.752^c^	—
Cognitive composite score, Median (IQR)	105 (95–111.25)	115 (100–122.5)	110 (100–120)	**0.014**^c^ **(ε**^**2**^** = 0.071)**	**VPT vs MLP: 0.006**^**b**^ VPT vs term: 0.029^b^ MLP vs term: 0.572^b^
> 85, *n* (%) Average and above	32 (84.2%)	49 (100%)	33 (97.1%)	**0.024**^a^	**VPT vs MLP: 0.016**^**a**^ VPT vs term: 0.149^a^ MLP vs term: 0.410^a^
70 to 85, *n* (%) Mild impairment	3 (7.9%)	0 (0%)	1 (2.9%)		
≤ 70, *n* (%) Moderate to severe impairment	3 (7.9%)	0 (0%)	0 (0%)		
Language composite score, Median (IQR)	98.5 (91–112.75)	112 (97–118)	103 (97–112.75)	**0.008**^c^ **(ε**^**2**^** = 0.081)**	**VPT vs MLP: 0.003**^**b**^ VPT vs term: 0.176^b^ MLP vs term: 0.073^b^
> 85, *n* (%) Average and above	35 (92.1%)	49 (100%)	34 (100%)	0.151^a^	—
70 to 85, *n* (%) Mild impairment	1 (2.6%)	0 (0%)	0 (0%)		
≤ 70, *n* (%) Moderate to severe impairment	2 (5.3%)	0 (0%)	0 (0%)		
Motor composite score, Median (IQR)	91 (84.25–100)	100 (91–110)	105 (94–112.75)	**0.001**^c^ **(ε**^**2**^** = 0.121)**	**VPT vs MLP: 0.004**^**b**^ **VPT vs term: < 0.001**^**b**^ MLP vs term: 0.211^b^
> 85, *n* (%) Average and above	27 (71.1%)	45 (91.8%)	32 (94.1%)	**0.010**^a^	**VPT vs MLP: 0.018**^**a**^ VPT vs term: 0.030^a^ MLP vs term: 0.693^a^
70 to 85, *n* (%) Mild impairment	7 (18.4%)	4 (8.2%)	2 (5.9%)		
≤ 70, *n* (%) Moderate to severe impairment	4 (10.5%)	0 (0%)	0 (0%)		

Bayley-III = Bayley Scales of Infant and Toddler Development-Third Edition, IQR = interquartile range, MLP = moderate-to-late preterm, VPT = very preterm

ε^2^ = epsilon-squared effect size (≥ 0.01 = small effect; ≥ 0.06 = moderate effect; ≥ 0.14 = large effect)

Bold values indicate statistically significant at the *p* < 0.05 level. Post-hoc pairwise comparisons were adjusted using Bonferroni correction (*p* < 0.017). Bold values indicate statistically significant results after correction

^a^Pearson chi-square test

^b^Mann-Whitney *U* test

^c^Kruskal-Wallis test

### Multivariable linear regression analyses

Multivariable linear regression analysis was performed to assess the factors that predict the Bayley-III motor composite score in preterm infants. The model included the following variables: MOS-R, gestational age, IVH grade III–IV (yes/no), and BPD (yes/no). The model was found to be significant (*F*(4, 82) = 13.507, *p* < 0.001; adjusted *R*^2^ = 0.368). The MOS-R (*β* = 0.478, *p* < 0.001) was identified as an independent predictor of the Bayley-III motor results while gestational age, IVH, and BPD were not significant predictors. There was no multicollinearity in the model (Supplementary Table 2).

## Discussion

This study found that the normal GMs rate increased from 23 to 28% as infants transitioned to the postterm period, while the CS GMs rate decreased. The normal fidgety movement rate was 85.7%, slightly less than the sum of normal GMs and PR GMs. The other important finding was that the GMOS-R and MOS-R of VPT infants were also lower than term infants. Although a difference in GMOS-R between VPT and MLP infants was observed at the unadjusted level, this did not remain statistically significant after correction for multiple comparisons, and should therefore be interpreted with careful consideration. MLP infants’ performance on cognitive, language, and motor outcomes was more advanced than that of VPT infants. Additionally, term infants demonstrated better motor development compared to VPT infants. Although significant differences exist between VPT and term infants for GMOS-R and MOS-R, the predominantly small to moderate effect sizes suggest that the clinical importance of these findings requires cautious interpretation.

Previous studies demonstrate that the quality of early spontaneous movements improves with age-related maturation [28, 39, 40]. Our results were consistent with these studies, showing an increase in the frequency of normal movement patterns and a numerical increase in GMOS-R. Several studies [28, 39] on very preterm and extremely preterm infants showed a longitudinal increase in GMOS. One multicenter study found [14] a decrease in 233 preterm/term infants. The varying clinical characteristics or perinatal risk factors, in addition to socioeconomic and healthcare factors, could explain the difference.

Similar to previous studies [15, 28, 31, 41], the most frequent movement pattern in the preterm or term periods and the postterm period was PR GMs (ranging from 57 to 85%). Previous studies have indicated that the relatively high prevalence of PR GMs in the preterm or term periods tends to be significantly normalized during the fidgety period in VPT infants [31], extremely preterm infants [42], and infants undergoing neonatal surgery [43]. The high rate of transition from PR GMs to normal fidgety movements can also be explained by ongoing brain maturation and the partial recovery of motor networks that were initially under stress but remained structurally intact [12]. In infants without severe and persistent brain injury or major comorbidities, this physiological maturation process may enable normal fidgety movements.

Einspieler et al. [15] reported that the GMOS-R of high-income countries was higher than that of upper-middle-income countries and lower-middle-income countries, and that the GMOS-R for upper-middle-income countries was found to be 33 for normal GMs, 18 for PR GMs, and 10 for CS GMs in recordings from the extremely preterm period to the postterm period. Our country, which is classified as upper-middle-income by the World Bank [44], exhibited normal GMs with slightly lower but PR and CS GMs had slightly higher GMOS-R in the preterm or term and postterm periods.

In addition to fidgety movements, the MOS-R provides detailed information about the movement and postural patterns of the infant [16]. It is evident that term-born infants have higher MOS-R than those born very or extremely preterm [19, 45]. Similarly, the MOS-R of term infants was only higher than VPT infants in our study. The MOS-R of MLP infants was higher in both previous studies [46, 47]. However, the current study did not find a difference in MOS-R between these groups. The reasons for this could be due to the diverse clinical characteristics of the infants, different parental adaptations, and the environmental conditions to which the children were exposed.

Early motor behaviors are a key representation of developmental processes including cognitive and behavioral processes [12]. While Robinson et al. [48] reported that GMOS accurately predicted motor outcomes at 1 year and 2 years of age in high-risk infants, recent studies [49–52] specifically highlight the MOS-R. Research by Kadam et al. [49] and Kwong et al. [50] indicated that the higher MOS-R were significantly associated with improved motor, mental, and cognitive performance in VPT infants at 1–2 years of corrected age. Kwong et al. [51] also indicated that the correlation remained similar even when the fidgety-movement subcategory of the MOS-R was removed. In another study, it was found that all MOS-R subcategories other than fidgety movements were correlated with the cognitive, language, and motor domains of the Bayley-III between 12 and 42 months of age in all infants, while at least two MOS-R subcategories (except fidgety movements) were found to be associated with all Bayley-III domains in preterm children [53]. The current study supported the literature on the relationship between MOS-R and motor development, and also demonstrated that even without the fidgety movements subcategory, MOS-R remained correlated with language, cognitive, and motor development at one year of corrected age.

The relationships found in our study with developmental parameters at one year of corrected age were stronger with the MOS-R result than with the GMOS-R result and in our regression model, the MOS-R exhibited the highest predictive value. This reflects the age-specific nature of GMA [8, 13] and suggests that MOS-R may provide a more accurate quantitative assessment of early spontaneous movements and may be more sensitive to non-CP developmental differences than global GMA assessments [51].

We acknowledge several limitations to consider. First, the missingness rates for Bayley-III differed between groups; however, attrition analyses indicated that this was independent of infant demographics, GMOS-R and MOS-R. Second, focusing on a clinically referred “at risk” sample may limit the generalizability of the findings to broader populations. Third, assessments at 1 year of age may have low predictive validity and Bayley-III itself may overestimate developmental functioning. Fourth, excluding infants unable to complete any of the GMA periods due to the longitudinal design could introduce potential selection bias. Finally, the limited sample size in some subgroup comparisons requires a cautious interpretation of the findings.

## Conclusion

Although there was no difference in GMOS-R between VPT and MLP infants in the preterm or term periods and postterm period, GMOS-R in the postterm period of VPT infants and MOS-R in the fidgety period of VPT infants were lower than those of term infants. VPT infants had lower Bayley-III outcomes than MLP infants in the cognitive, language, and motor domains, and lower motor outcomes than term infants. MOS-R had a stronger relationship with cognitive, language, and motor development at one year than GMOS-R in all preterm infants, based on existing relationships. GMOS-R may support early risk stratification in the earliest months of life and is potentially informative for early intervention referrals; however, it should be interpreted with neuroimaging findings and neurological examination in the clinical decision-making process. Additionally, MOS-R demonstrates stronger overall associations than GMOS-R and is an independent predictor of the Bayley-III motor score in regression analysis, suggesting its valuable role in early developmental assessments.

## Supplementary information

Below is the link to the electronic supplementary material.

ESM 1 (DOCX 18.3 KB)

ESM 2 (DOCX 18.6 KB)

## Data Availability

No datasets were generated or analysed during the current study.
