# Supplementary material for: Trajectories of detailed general movements and their association with one-year developmental outcomes in very preterm, moderate-to-late preterm, and term infants
Source: Eur J Pediatr. 2026 May 1;185(5):330. doi: 10.1007/s00431-026-06971-x (PMC13135000; doi:10.1007/s00431-026-06971-x)
Supplement: Supplementary file 1 — (DOCX 18.3 KB) [file 431_2026_6971_MOESM1_ESM.docx]

**Supplementary Table 1.** The correlations between early spontaneous movements and Bayley-III subdomains in preterm infants

| **Early Spontaneous Movements** | **Bayley-III Subsections** | | | | | |
| --- | --- | --- | --- | --- | --- | --- |
|  | **Cognitive Domain** | | **Language Domain** | | **Motor Domain** | |
|  | **r** | **p** | **r** | **p** | **r** | **p** |
| ***Preterm or term period*** |  |  |  |  |  |  |
| GMOS-R | .171 | 0.113 | .096 | 0.378 | **.329** | **0.002** |
| Global GMs (CS, PR or N) | .146 | 0.177 | .201 | 0.063 | **.255** | **0.017** |
| ***Postterm period*** |  |  |  |  |  |  |
| GMOS-R | **.222** | **0.039** | **.215** | **0.045** | **.340** | **0.001** |
| Global GMs (CS, PR or N) | **.355** | **0.001** | .179 | 0.098 | **.363** | **0.001** |
| ***Fidgety period*** |  |  |  |  |  |  |
| MOS-R | **.396** | **<0.001** | **.301** | **0.005** | **.457** | **<0.001** |
| MOS-R without fidgety movements subcategory | **.393** | **<0.001** | **.281** | **0.008** | **.394** | **<0.001** |
| Fidgety movements subcategory | **.293** | **0.006** | **.257** | **0.016** | **.451** | **<0.001** |

GMs= General movements, GMOS-R= General Movement Optimality Score–Revised, MOS-R= Motor Optimality Score-Revised, N: Normal, PR: Poor repertoire, CS: cramped synchronized

r: Spearman’s correlation coefficient

Bold values indicate statistically significant at the p < 0.05 level.
