# Supplementary material for: Trajectories of detailed general movements and their association with one-year developmental outcomes in very preterm, moderate-to-late preterm, and term infants
Source: Eur J Pediatr. 2026 May 1;185(5):330. doi: 10.1007/s00431-026-06971-x (PMC13135000; doi:10.1007/s00431-026-06971-x)
Supplement: Supplementary file 2 — (DOCX 18.6 KB) [file 431_2026_6971_MOESM2_ESM.docx]

**Supplementary Table 2.** Multivariable linear regression analyses of the predictors for the Bayley-III motor domain composite score

| **Independent Variables** | **B** | **SE** | **Beta (β)** | **t** | **p** | **95% CI for B** |
| --- | --- | --- | --- | --- | --- | --- |
| Constant | 46.57 | 17.71 | - | 2.63 | **0.010** | 11.34 to 81.80 |
| MOS-R | 1.15 | 0.22 | 0.47 | 5.12 | **<0.001** | 0.70 to 1.60 |
| Gestational age | 0.37 | 0.57 | 0.07 | 0.64 | 0.520 | -0.77 to 1.51 |
| IVH Grade III–IV | 4.08 | 9.04 | 0.04 | 0.45 | 0.653 | -13.91 to 22.07 |
| BPD | 8.90 | 4.50 | 0.22 | 1.97 | 0.052 | -0.065 to 17.86 |
| Model statistics: Adjusted R² = 0.368, F(4, 82) = 13.507, p<0.001  VIF values among the respective variables ranged from 1.118 to 1.774 | | | | | | |

B: Unstandardized regression coefficient, β: Standardized regression coefficient, Bayley-III= Bayley Scales of Infant and Toddler Development-Third Edition, BPD= Bronchopulmonary dysplasia, IVH= Intraventriculer hemorrhage, MOS-R= Motor Optimality Score-Revised, SE: Standart error. VIF= Multicollinearity

Bold values indicates statistically significant at the P < 0.05 level.
